# Letter to the editor about the article “relationship between menopausal hormone therapy and incidence risk of breast cancer: systematic review and meta-analysis”

**DOI:** 10.1080/07853890.2026.2654102

**Published:** 2026-04-06

**Authors:** Anne Gompel

**Affiliations:** Department of Gynecology, Université Paris Cité, Paris, France

Dear Editor

We read with interest the article from Wu Q. et al. entitled “Relationship between menopausal hormone therapy and incidence risk of breast cancer: systematic review and meta-analysis” [1]. We were very surprised that many studies addressing the same question, i.e. is breast cancer risk associated with Menopause Hormone Therapy, were not included in the meta-analysis, despite being cited, such as the study by Vinogradova [2] (present in the list as ref. 76), but not included in the tables, as the studies from Fournier et al. [3,4]. Furthermore, the Women’s Health Initiative Randomised Controlled Trials have been included in the analysis several times, even though they refer to the same populations. Can the authors explain the rationale of their choices by selecting the 34 studies from a much larger dataset, repeating the inclusion of the same populations, and excluding major studies?

Despite the thorough analysis conducted in this article, we think that excluding large studies and repeatedly including the same populations might have introduced important bias into the conclusions.

Sincerely

Pr. Anne Gompel

## Data Availability

No new data were generated or associated with this manuscript.
